# Validations of various in-hand object manipulation strategies employing a novel tactile sensor developed for an under-actuated robot hand

**DOI:** 10.3389/frobt.2024.1460589

**Published:** 2024-09-26

**Authors:** Avinash Singh, Massimilano Pinto, Petros Kaltsas, Salvatore Pirozzi, Shifa Sulaiman, Fanny Ficuciello

**Affiliations:** ^1^ Department of Information Technology and Electrical Engineering, Università degli Studi di Napoli Federico II, Napoli, Italy; ^2^ Department of Engineering, Università degli Studi della Campania “Luigi Vanvitelli”, Caserta, Italy

**Keywords:** under actuation, in-hand manipulation, tactile sensing, neural networks architectures, control

## Abstract

Prisma Hand II is an under-actuated prosthetic hand developed at the University of Naples, Federico II to study in-hand manipulations during grasping activities. 3 motors equipped on the robotic hand drive 19 joints using elastic tendons. The operations of the hand are achieved by combining tactile hand sensing with under-actuation capabilities. The hand has the potential to be employed in both industrial and prosthetic applications due to its dexterous motion capabilities. However, currently there are no commercially available tactile sensors with compatible dimensions suitable for the prosthetic hand. Hence, in this work, we develop a novel tactile sensor designed based on an opto-electronic technology for the Prisma Hand II. The optimised dimensions of the proposed sensor made it possible to be integrated with the fingertips of the prosthetic hand. The output voltage obtained from the novel tactile sensor is used to determine optimum grasping forces and torques during in-hand manipulation tasks employing Neural Networks (NNs). The grasping force values obtained using a Convolutional Neural Network (CNN) and an Artificial Neural Network (ANN) are compared based on Mean Square Error (MSE) values to find out a better training network for the tasks. The tactile sensing capabilities of the proposed novel sensing method are presented and compared in simulation studies and experimental validations using various hand manipulation tasks. The developed tactile sensor is found to be showcasing a better performance compared to previous version of the sensor used in the hand.

## 1 Introduction

Nowadays, replacement of a missing limb due to trauma, illness, or any congenital conditions present from birth with prosthetic upper limbs is a significant challenge to the medical industry. This has prompted a need to create intelligent and multi-functional prosthetic hands as a means of replacing an amputated limb of a patient. Many aspects of prosthetic hand including appropriate weight, ergonomic design, easiness of use, stable force feedback, sufficient grasping, and manipulation capabilities should be taken into account in order to meet a patient’s needs. When the aforementioned factors are in proper proportion, a prosthetic device can assist a patient to perform daily activities effortlessly.

Autonomous in-hand object manipulation capabilities are the most sought-after essential abilities for the prosthetic hands (Feigl et al., 2023). Numerous studies have been going on in the field of development of prosthetic devices controlled by a patient’s electromyographic (EMG) data (Park et al., 2024; Liu et al., 2024). Along with EMG data, inclusion of touch sensors into a prosthetic device can manipulate objects in various positions and orientations by controlling grasping forces (Sharma et al., 2023). Touch sensors currently used in prosthetic hands have some drawbacks that limit their effectiveness. One major issue is their lack of sensitivity which results in inaccurate detection of amount of grasping forces. Another drawback is the durability of these sensors as they can wear out quickly with regular use. This can lead to the need for frequent replacements which can be costly and time-consuming. Additionally the size and weight of the sensors can also be a limitation as they may add bulk to the prosthetic hand and make it less comfortable for the user to wear for extended periods of time. One of the major limitation related to touch sensors employed in finger tips is their dimensional incompatibility. Developing a compatible touch sensor for fingertips is a challenging task due to the dimensional constraints. Incompatible touch sensors equipped on fingertips will result into inaccurate grasp force values. Hence these limitations highlight the need for continued research and development in improving the functionality of touch sensors in prosthetic hands to better mimic the capabilities of a human hand.

This paper addresses the current shortcomings of available sensing methods of prosthetic hands and proposes a novel tactile sensor developed based on opto-electronic technology for a prosthetic hand named “Prisma Hand II.” Our work aims to develop a dimensionally compatible tactile sensor to integrate with the fingertips of the Prisma Hand II. We managed to create a 3×3 tactile map in the constrained fingertip area and determined grasping forces and torques. The developed sensor is durable, light weight, cost effective, and accurate in predicting force and torque values during grasping task. Neural Networks (NNs) are utilized to estimate the ideal gripping forces during in-hand manipulation tasks based on the output voltage acquired from the novel tactile sensor. Two types of NNs were compared to determine a better training algorithm to obtain grasping forces during manipulation tasks. We illustrate how to use reconstructed forces and voltages from touch sensors to perform in-hand manipulation challenges. We utilized and integrated taxels voltages for manipulating objects in presence of external disturbances. The sensing capabilities and performance of the sensor are demonstrated using simulation studies and experimental validations.

## 2 State of art

Manipulation of an object using an under-actuated hand is a challenging yet promising field of research that continues to push the boundaries of robotic manipulation capabilities. By developing innovative control strategies and improving the design of hands, researchers aim to enhance the versatility and efficiency of robotic manipulation systems. As technology advances, object manipulation is expected to play a key role in various industries, revolutionizing the way tasks are performed in complex and dynamic environments. There are various kinds of prosthetic hands currently available in the market suitable for hand disabled patients (Belter et al., 2013; Van Der Niet and van der Sluis, 2013; Marateb et al., 2021; Liu et al., 2019). Most of these prosthetic hands are employed with tactile sensors to manipulate the physical interactions. Tactile sensors work based on changes in the values of resistance, capacitance, optical distribution, and electrical charges (Fraden and King, 2004; Russell, 1990). Piezoresistive (Almassri et al., 2015), Piezoelectric (Nassar et al., 2023), capacitive (Ge et al., 2022), quantum tunnel effect (Shi et al., 2020), optical (Fujiwara et al., 2018), and barometric (Liu et al., 2023) sensors are the most common types of tactile sensors used in prosthetic hands.

Contact point locations of a fingertip are determined using reconstructed forces and torques values obtained from a tactile sensor integrated to a hand in Liu et al. (2012b). The measured force values can be used for carrying out force controlling strategies and optimising grasping forces. Fast responding tactile sensors such as piezoelectric, capacitive, and barometric sensors can measure vibrations at contact points. The vibration information can be used for detection of slip and object exploration. Tactile sensors are capable to measure object shape and pressure distributions at contact points (Kyberd and Chappell, 1992). Capacitive and piezoresistive sensors can gather data from taxels directly using multiplexing circuits. Increasing the number of tactile sensors in a fingertip can lead to issues with wiring. These issues can be overcome by using plastic optical fibres in the tactile sensors. A 2 layered silicone based haptic tactile sensor is proposed in Sato et al. (2008). The displacement of the marker is recorded by a camera and can compute the magnitude of the force with a resolution of 0.3 N. An optical fibre based sensor named Fiber Bragg grating (FBG) senso embedded in a silicone based elastomer is introduced in Heo et al. (2008). The sensor can detect forces upto 15 N with a resolution of 0.05 N. The main drawback of the sensor is the presence of hysteresis loss due to silicone material embedded to the sensor. Microbending optical fiber (MBOF) sensors are also demonstrated in Heo et al. (2008) to reduce the effect of optical bending issues. Another tactile optical sensor with a silicone based skin equipped to a fingertip is given in Yamada et al. (2005). Reflector chips made up of steel acts as the top layer of the silicone skin. The intensity and direction of applied forces are measured using optical sensing method. In order to avoid the bulky nature of the optical fibres, LEDs are introduced into the tactile sensors in Rossiter and Mukai (2005). The sensor showcased 4×4 matrix structure with a sensitivity in the range of 1 mV/N. Another LED based tactile sensor with 8×4 array is presented in Ohmura et al. (2006). The sensor is composed of a flexible polyurethane foil, that can bent to adapt to complex curved surfaces. A tactile sensor with 2 LEDs stacked on top of each other and placed beneath an elastic membrane is demonstrated in Hoshino and Mori (2008). When a contact force is applied, the pattern of light under the membrane changes and position along with magnitude of force can be measured using the technique.

Pressure profiles obtained during application of force on a tactile surface are generated to measure force values in Weiß and Worn (2005). The pressure profiles are mostly used for recognising contacts (Liu et al., 2012a), estimating grasp intensity (Bekiroglu et al., 2011), and classifying objects (Drimus et al., 2014). Integrating tactile sensors to fingers and fingertips is a complex task due to dimensional constraints. Tactile sensors can be flexible (Schmitz et al., 2010) or rigid (Koiva et al., 2013) based on the types of surfaces and required outputs. Different types of sensors equipped on robotic hands and their characteristics are given in Supplementary Table 1.

Machine learning techniques are currently being used in a number of studies for determining forces acting at contact points using tactile sensing systems. Feature extraction is performed to filter raw signals and decrease the computational time by reducing transmission of unwanted data through tactile sensing system (Dahiya et al., 2013). Unsupervised way of learning is applied to recognise features from a tactile sensing system. In Madry et al. (2014), an unsupervised feature learning scheme is employed to extract features from raw images and pool these images to determine grasping force. Along with unsupervised learning methods, deep learning techniques are also used for extracting features from tactile sensors. Krizhevsky et al. (2012) adopted a CNN to extract features of a tactile image and Sohn et al. (2017) employed a Recurrent Neural Network (RNN) to analyse the deformation of the tactile sensor padding. Most of the learning based feature extraction methods are prone to over-fitting issues. In order to reduce the probability of over-fitting, most learning methods are accompanied by feature selection methods (Servati et al., 2017). Bhattacharjee et al. (2012) proposed a K-Nearest Neighbour (KNN) strategy to recognise contact points using a tactile sensor. However, computational time is higher during contact point recognition compared to traditional recognition methods. Support vector machines (SVM) methods are employed along with tactile sensors to determine contact points, and grasping forces. Gastaldo et al. (2014) employed SVM method to obtain contact point locations during a grasping activity. Even-though the SVM method performed better showcasing high accuracy predictions, computational effort is higher during the prediction process.

Nowadays, researchers are focusing more on deep learning techniques to increase the performances during predictions. Deep learning techniques have been successfully outperformed most of the conventional machine learning methods (Zou et al., 2017; Zhao et al., 2018). Deep Neural Network (DNN) based force reconstruction from image patterns are presented in Sferrazza et al. (2019). The proposed DNN performed well during real time testing, however, generalization capabilities of the strategy are not analysed. Radical Basis Function Neural Network (RBFNN) based prediction of contact point forces are carried out in Wang and Song (2021), Zhang et al. (2018). RBFNN exhibited greater generalization capabilities by linearising non linear functions with high precision. However, real time prediction of 3D forces are not achieved using the technique. Correlating sensor signals using ANN based approaches is given in Gao et al. (2018), Chuah and Kim (2016). The Artificial Neural Network (ANN) based networks performed better during simulation studies and however, computational effort is higher during real—time implementations. Electrical Impedance Tomography Neural Network (EIT-NN) based low-cost sensor is given in Park et al. (2021). Convolutional Neural Network (CNN) based tactile systems are employed in Kakani et al. (2021) and Meier et al. (2016) to determine force and motion characteristics during a grasping task. Various machine learning integrated tactile sensing systems used in different applications are given in Supplementary Table 2.

Response timing of tactile sensors is crucial during a task execution. Fast and accurate sensing capabilities will increase the efficiency of a task execution. A tactile sensor manufactured using polystyrene microspheres (Li et al., 2016) exihibited a fast response time of 38 ms. A piezoresistive tactile sensor (Wang et al., 2019) consisted of Galinstan micro-channels has a response time of 90 ms. A flexible pressure sensor (Wang et al., 2019) composed of PZT nano ribbons exhibited a fast response rate of 0.1 ms. Chun et al. (2016) proposed a tactile sensor manufactured using a conductive graphene-sponge composite. The response time of the proposed sensor is obtained as 5 ms. Several actuation techniques have also been referenced such as elastic actuation in form of a series of elastic tendons (Grebenstein et al., 2012) combined with compliant links made up of steel layers (Choi et al., 2017). Also several attempts for flexure based (Odhner et al., 2014) and spring-based (Lotti et al., 2005) elastic compliant finger joints are made. Several robotic hands such as fully-actuated hands like Shadow Dexterous Hand (Andrychowicz et al., 2020), KITECH hand (Lee et al., 2016) and BCL-13 (Zhou et al., 2018) have been developed based on individual joint control.

Controller schemes adopted for manipulating the hand postures are crucial to carryout a grasping task effectively. In Ficuciello et al. (2012), 3 postural synergies based control strategies are adopted for improving the efficiency of grasp executions carried out by a UB Hand IV (University of Bologna Hand, version IV). Smooth hand motions are obtained using the proposed control strategy during experimental validations. A study has been conducted in Ficuciello et al. (2018) for investigating the effectiveness of postural synergies employed in an anthropomorphic hand named SCHUNK 5-Fingered Hand (S5FH). Inverse kinematic schemes and feedbacks from an RGBD camera are used for manipulating the motions of the hand. The motor current values are utilized to restrict the grasping forces using the motor position control in the synergy subspace. An another synergy based control strategy adopted for the S5FH is demonstrated in Ficuciello (2018). Synergy parameters are computed based on an under-actuated kinematic approach. The proposed control strategy is composed of a feedforward and 2 correction parameters to determine fingertip positions during grasping. The development of a reinforcement learning algorithm for an anthropomorphic hand-arm system using policy search methods is given in Ficuciello (2019). The reward function generated during the learning is used to assess the effectiveness of the grasp in a synergy-based framework. Experiments are conducted on a KUKA LWR4+ manipulator equipped with a S5FH to demonstrate the effectiveness of the method. Optimal contact forces are computed in Villani et al. (2012) considering the contact points and wrench. Feedback from tactile and force sensors are used for obtaining an optimal grasp.

In this paper, a novel tactile sensing method developed for enhancing grasping capabilities of a prosthetic hand named “PRISMA Hand II” based on opto-electronic technology is presented. In this work, we chose an optical tactile sensor for the prosthetic hand, since optical tactile sensors are considered as the most durable and flexible sensors with high spatial resolution (Ascari et al., 2007). We developed a compatible tactile sensor and successfully integrated to the fingertips of the prosthetic hand.

We calibrated the sensor using a reference sensor (ATI) in order to train NN to reconstruct contact force components. As a result, the same sensor provided a tactile map and contact force estimation at the same time, by allowing us to implement different control approaches with the same device. Grasping force and torque values in 3 axes to manipulate an object are determined separately using ANN and CNN training methods. The accuracy of force values obtained using these 2 NNs is compared using Mean Square Error (MSE) values. An In hand manipulation control technique for grasping activities of an object using hand is also developed. The control scheme computed grasp forces/torques based on the reconstructed data from sensors. Additionally, it also controlled the orientation of the object using taxel voltage data. Experimental validations are carried out to study the performance of the proposed strategies.

## 3 Design and sensing system of the PRISMA Hand II

**FIGURE 1:**
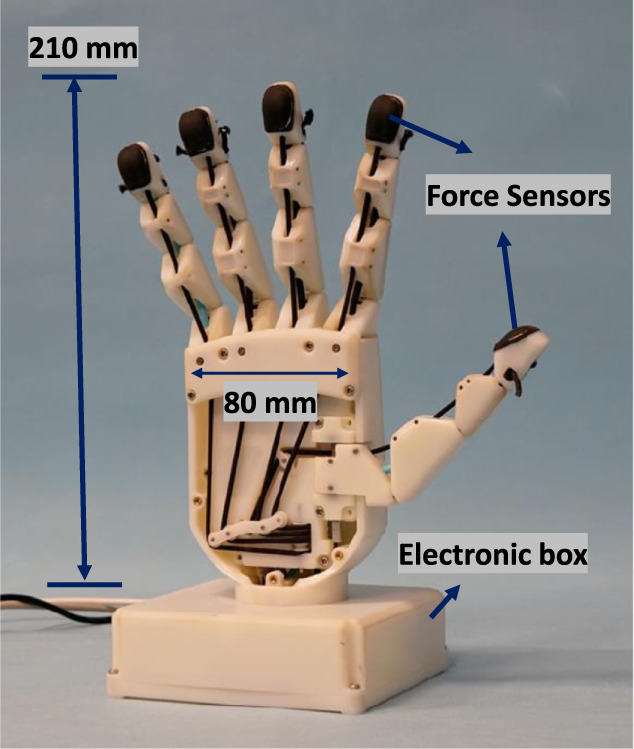
Prisma Hand II (Ficuciello et al., 2020).

PRISMA Hand II (shown in Figure 1), an updated model of PRISMA Hand I (Ficuciello et al., 2020) is a 3D printed tendon-actuated prosthetic hand made up of 19 joints driven by 3 motors and weighs around 0.339 kg. The thumb is composed of 1 revolute joint and 3 flexion/extension compliant rolling joints. The index, ring, and little fingers are consisted of a single revolute joint and 3 flexing/extension joints. It comprised of fingertip tactile sensors to compute voltage/force feedback.

**FIGURE 2:**
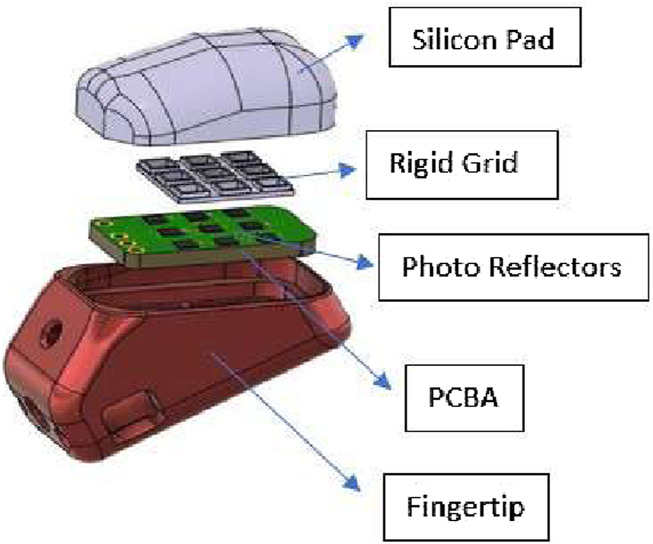
Prisma Hand II fingertip sensor.

A novel tactile sensing technology was developed for the PRISMA Hand II to improve manipulation capabilities of the hand during grasping. The tactile sensing method employed a taxel sensing method based on optoelectronic technology (Cirillo et al., 2017). The sensor shown in Figure 2 operated based on the change in intensity of reflected light caused by pressing the fingertip deformable pads, which was then measured by photocouples and output a voltage value. Depending on the deformation of the pad above the phototaxels, the reflected light on the photocouples changed the photocurrent values. The change in values of photocurrent caused a voltage change that was detected by an Analog to Digital Converter (ADC) on the printed circuit board (PCB). The output of the ADC converter was obtained in the form of digital bytes. Voltage output bytes were translated to force and torque values in the 3 axes (X,Y,Z) using two NNs training methods such as ANN and CNN methods. MSE values were used to determine the highest performing network. During the calibration procedure, a 3 axes ATI F/T sensor served as a force/torque reference to establish the relationship between voltage and force.

**FIGURE 3:**
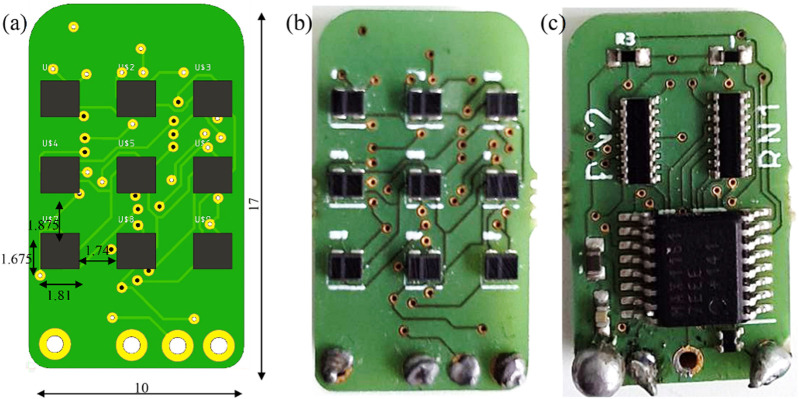
Fingertip sensor **(A)**Dimensions (in mm). **(B)**Front view. **(C)**Rear view.

The dimensions of the fingertip sensor along with front and rear views are shown in Figure 3. A PCB containing LED-photo transistor couples was covered by an elastic pad. The deformable pad consisted of a top layer of a black silicon (MM928) layer with an opaque black grid sandwiched between the silicon and the PCB. This prevented light disturbances and non-monotonic behavior under high stresses, guaranteeing maximum reflection of light at all wavelengths. The ability to receive light of any wavelength can improve the sensitivity and precision of the sensors. 2 Capacitors (C3 (0.1 uF) Ceramic and C4 (4.7 uF) Tantalum) and resistors (R2 (270 kΩ), R3 (3.9 kΩ), RN1 (3.9 kΩ), RN2 (270 kΩ), and RN4 (1 kΩ)) equipped on the PCB aided the conversion of photocurrent to voltage during operation of the sensors.

The sensor previously employed on the hand was composed of a 2 × 2 matrix structure photo transistor couples (2 × 2) placed on top of the PCB. The sensor employed an ADC converter [ADS1015 (4 channels)]. The sensors were trained using NNs utilizing force readings from the ATI F/T sensor. On the contrary, the new optimised sensors developed in this work consisted of a 3 × 3 matrix structure photo transistor couples. The improvised sensor was connected to an Arduino Nano micro controller using an I2C connection protocol. Data were communicated in the form of bits during the operation of sensors. The unique feature of the new sensors allowed us to use a combination of force and torque data from an ATI sensor as an input for training a NN. This decreased MSE errors and improved precision of force prediction. The increased amount of input data samples (force + torque) used to train the NNs accounted for the higher precision in output force prediction. Furthermore, the bits were converted to a voltage, V with respect to a reference voltage, $V_{\mathrm{ref}}$ using the below mentioned formula:

$$V=\frac{V_{\mathrm{ref}}\times Bytes}{4096}$$

For the sensors given in the study, the $V_{\mathrm{ref}}$ value was set to 3.3V from the Arduino microcontroller. The ADC converted bytes to digital values. The selected ADC type was MAX11617, with 12 channels, resulting in 4,096 ( $2^{12}$ ). The upgradation of the ADC channels from 4 to 12 allowed us to include torque measurements in addition to force during calibration. The connection protocol comprised of four wires: SDA (serial data), SCL (serial clock), input voltage (3.3 V), and ground. To quantify all the 5 fingertip sensors together, a multiplexer/I2C expander (TCA9548A) was linked as a bridge between sensors and Arduino micro controller. This provided a solution for addressing the 5 fingers simultaneously using a single electrical board. It is important to note that these performances must be evaluated in light of the fact that, when compared to commercially available sensors, the proposed one is more compact, less expensive, consumes less power, has a digital interface, and the deformable layer ensures good adaptability and stability during grasping and manipulation applications.

## 4 Force mapping using neural networks

**FIGURE 4:**
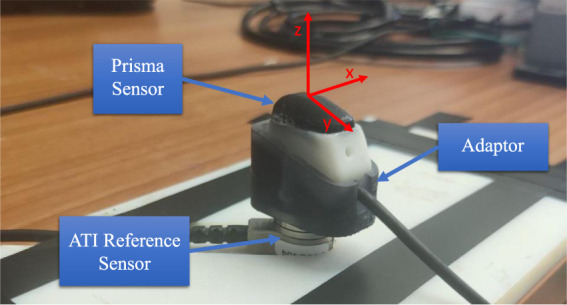
Setup for calibration of sensor.

The process of force mapping was performed to establish a relationship between the output voltage from the sensor and the reference force/torque values in 3 axes (X, Y, Z) from an ATI F/T sensor by means of ANN and CNN networks separately. The fore-mentioned relationship was established during a calibration process by recording 140,000 samples of voltages and relative forces at a frequency of 100 Hz using ROS (Koubâa, 2017). During the calibration process, the pad was pressed and moved at various angles to capture as many data samples as possible using a grasping procedure. An ANN network developed in Cirillo et al. (2017) was used for training the data samples. TensorFlow and Keras sequential models with dense and activation layers were used for training on a Python framework. The performance of NN models was compared based on accuracy of force/torque predictions obtained after training. The parameter for selecting the optimal model was based on the lowest MSE value in the force/torque prediction phase of tests. Figure 4 depicts sensor calibration using the axis frame of reference.

**FIGURE 5:**
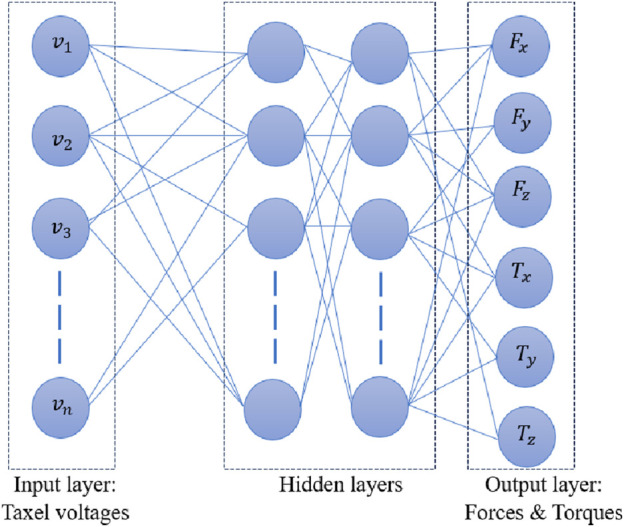
ANN architecture.

An ANN network trained using Levenberg—Marquardt (LM) method is depicted in Figure 5. Input layer was composed of output voltages from various taxels and forces/torques in 3 axes comprised the output layer. The properties of hidden layers were varied based on the task and provided in the coming sections.

**FIGURE 6:**
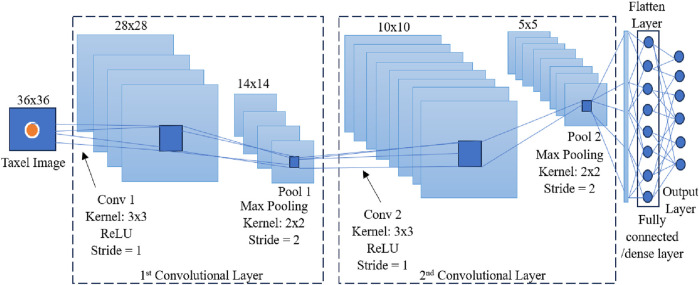
CNN architecture.

A CNN network was also used for training the data samples to reconstruct force/torque values. CNN architecture used in this work is given in Figure 6. Taxel image extractions were performed using two convolutional layers as shown in the figure. ReLU function was used as the activation function in the convolutional layers. A 3 × 3 kernel with stride value 1 and padding value 1 was used in convolutional layers accompanied by a pooling process with 2 × 2 kernel, stride value 2 and padding value 1. Fully connected/dense layers of NNs were used for predicting force and torque values from the extracted features from the convolutional layers.

## 5 In-hand manipulation control

In hand manipulation control method was employed to obtain an optimum grasp on objects during object manipulation tasks. The objects were manipulated based on voltages (raw data) received from each sensor taxel. Force/torque values were reconstructed using NNs (ANN and CNN networks) separately and evaluated to determine optimum grasps.

The operating principle of in-hand manipulation was based on correct voltage thresholding of all 9 taxel values collected from the sensor. The fingers were capable of executing sufficient motions to ensure a successful grasp and manipulation of objects to avoid grips on objects. The key principle of threshold selection was based on a real-time computation of the average values of all taxels’ voltages, $v_{a}$ which was then multiplied by a constant factor, K as mentioned below:

$$v_{t}=K\times v_{a}$$

The adoption of real-time mean voltage readings was critical since the sensor was extremely sensitive and not possible to be set to a conventional threshold value. The contact points between fingertip and object can change during a grasping task. This causes a shift in the pressure points of the pad and result in a varying voltage throughout the task. The second rationale for using the average was to account for voltage variations across all taxels and examine the whole contact surface rather than just one specific location. A heuristic approach was taken for selecting the constant of proportionality K, as changes in K can significantly affect the spectrum of acceptable and non-feasible contact. In theory, increasing the K value reduces the range of safe contact, whereas decreasing the K value reduces the range of unsafe contact. The value of K was determined by conducting multiple tests and observing the behavior of voltage change. The threshold value was then compared to the real taxel voltage measurements. The conditions in which the taxel voltage falls below the threshold were regarded as unsafe contacts. A taxel voltage equal to or greater than the threshold was considered as a safe contact between an object and a component of the taxel-related sensor. The objective was to consider the contact unsafe when a minimum number of taxels (an entire row) obeys the unsafe contact point conditions. The steps followed during in-hand manipulation control scheme is given in Algorithm 1.

Algorithm 1 In hand manipulation scheme.

**Input**:
1. Voltages obtained from all taxels,

$v_{i},i=1,2,\ldots 9$

**Output**: Optimum value of grasping contact and force:
2. Initialise the parameter sets and start grasping task
3. Compute average voltage values, $v_{t}$

4. Compute constant of proportionality, K

5. For all values of $v_{i}$ , calculate voltage threshold, $v_{t}$ ;

$v_{t}=K\times v_{a}$

6.
**if** $v_{t}\leq v_{i}$ **then**
 7. final voltage, $v_{f}=v_{i}$

 8. Grasping contact and force finalised
 10.
**else**
 11. Update the position of grasp and go to step 5
 12.
**end if**

## 6 Results and discussion

In this work, experimental validations on sensors equipped on the thumb of the Prisma hand II were carried out to analyse performance of the developed sensor. Simulation studies and experimental validations were performed using different architectures of CNN and experimentation set ups. The thumb of the hand plays a major role in the horizontal and vertical in-hand manipulations. Hence, thumb was selected to study the performance of the sensor developed for the hand.

### 6.1 Experimental validation of working of sensors

ANN and CNN networks were used for predicting 3 axes force values in X, Y, and Z directions. Voltages transformed into a 3 × 3 matrix structure were given as the input for training networks. A sigmoid function was used as an activation function during ANN training process. A batch size of 64 samples and 300 epochs were used for training ANN. The whole sample of data was divided into 80 percent for training and 20 percent for validating the network. Adaptive Moment Estimation (ADAM) was used to optimise the results and respective MSE values of the ANN trained in the X, Y, and Z-axes were found to be MSEx = 0.053, MSEy = 0.049, and MSEz = 0.319 respectively. To compare and select a best performing network, a CNN was also used for training the same dataset. The CNN comprised 2 convolutional layers of 256 and 64 neurons in addition to a dense layer of 16 neurons. The dataset was divided into 80 percent of training data and 20 percent of validation data. CNN used a batch size of 64 and epochs of 300. The MSE values for CNN trained in the X, Y, and Z-axes were found to be MSEx = 0.022, MSEy = 0.022, and MSEz = 0.148 respectively.

**FIGURE 7:**
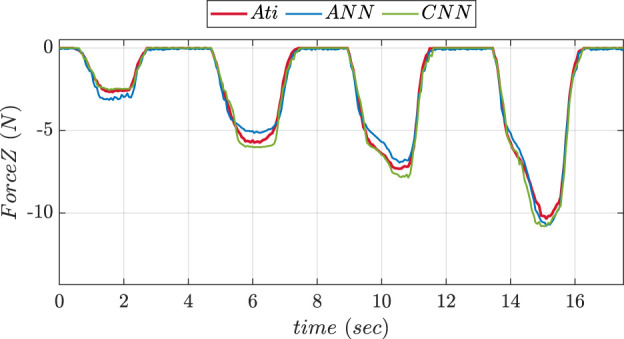
Comparative prediction graph of Z-axis force using ANN and CNN during push experiment.

**FIGURE 8:**
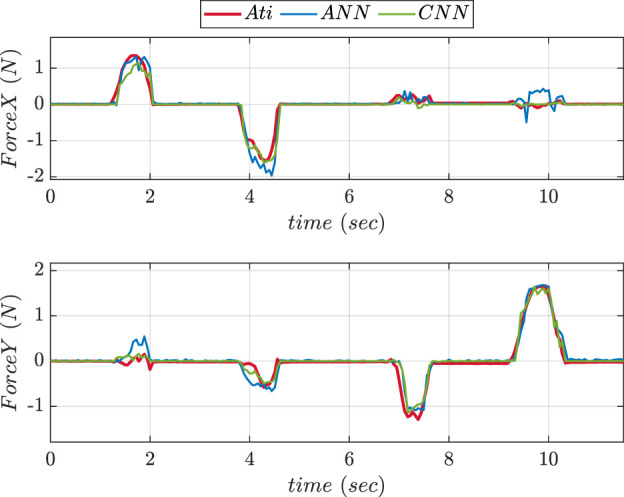
Comparative prediction graphs of X and Y-axes force using ANN and CNN during slide experiment.

To compare the performance of two NNs, experiments were conducted in 2 phases using the fingertip pad carrying out a pushing and a sliding tasks. During pushing tasks, NN was supposed to be precisely predicting the Z axis forces affecting the ATI reference sensor. The NN was assumed to be anticipating X and Y-axis forces acting on the object during sliding task. Figure 7 depicts the graphs comparing the force values in Z-direction predicted using ANN and CNN trainings during pushing experiment. The graphs comparing the predicted values of forces in X and Y directions during sliding experiment using ANN and CNN trainings are shown in Figure 8. The MSE values for prediction of force in Z axis using ANN and CNN during pushing experiment were found to be 0.2164 and 0.101 respectively. MSE values of forces in X direction during sliding experiment were found to be 0.016 and 0.013 respectively. The Y-axis forces during the inference/prediction phase of sliding experiment for ANN and CNN were found to be 0.024 and 0.010 respectively. The lowest MSE value corresponded to the best NN choice.

**FIGURE 9:**
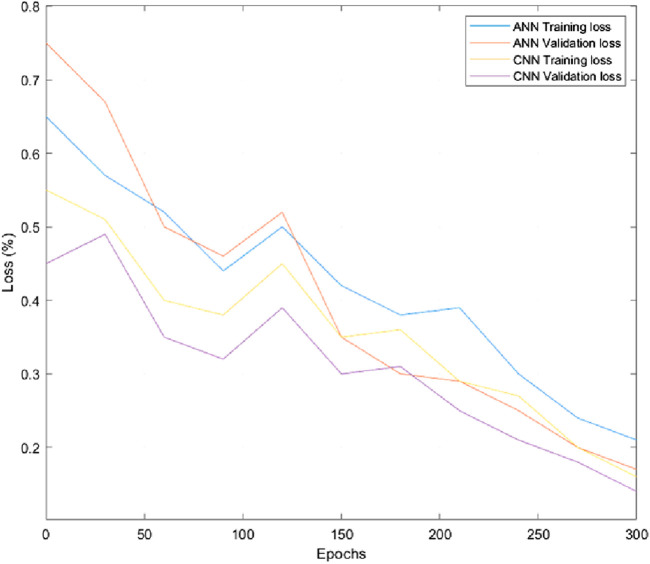
Training and validation losses during simulation.

**FIGURE 10:**
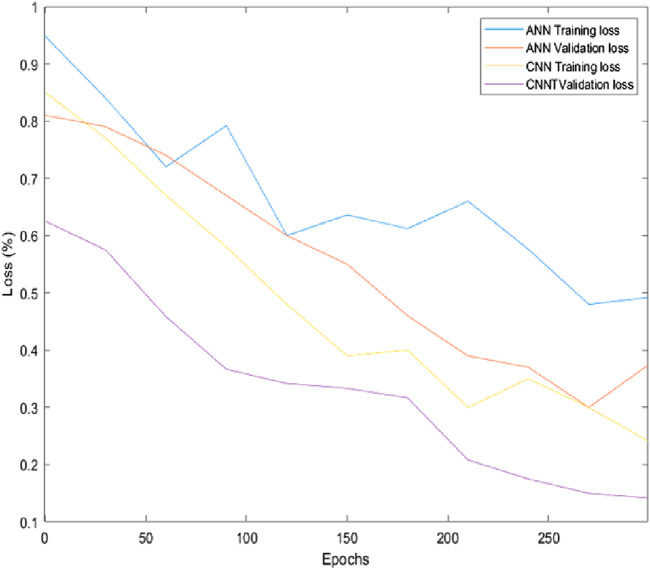
Training and validation losses during experimentation.

Figures 9 and 10 depict the comparison of training and validation losses during simulation and experimentation validations respectively. The training and validation losses computed using ANN and CNN networks at epoch 300 during simulation were obtained as 21%, 17%, 15%, and 13% respectively. Similarly training and validation losses during experimentations using ANN and CNN were obtained as 40%, 37.5%, 24% and 17% respectively. Hence, it is evident that CNN performed better in terms of losses for the specified task compared to ANN. The parameters and outcomes of ANN and CNN training networks during simulation studies and experimentations are given in Supplementary Tables 3, 4.

The accuracy of the ANN model seems to be more stable when momentum is utilized, exhibiting less volatility during the training epochs. Better performance is achieved with smaller decay levels. With a decay of $1\times 10^{-3}$ , the final learning rate for ANN training is computed to be around 0.001. CNN model performed showcased a learning rate of 0.0001 and accuracy above 90%. Hence, we can conclude that CNN performed better than ANN during simulation studies and experimentations due to lower MSE values, higher accuracy, higher learning rate, less training loss, and validation loss compared to ANN in the prediction phase. The model trainings are showcasing an underfitting state for both ANN and CNN based methods as evident from Figures 9, 10. Our primary aim was to compare the performances of two types of NNs within 300 epochs (we set 300 epochs to limit the computational effort). After the initial phase of comparison study, we understood that CNN performed better within the stipulated time with higher accuracy, lower training, and validation losses. We continued using the CNN training method by increasing epochs along with adding more data samples and more layers in the succeeding experiments to eliminate under-fitting/over-fitting issues and to achieve more accuracy.

### 6.2 Experimental validation using updated convolutional neural network

#### 6.2.1 Inclusion of torque values in CNN training

The previous approach to train a CNN was based on the inclusion of only 3 axes force values. To evaluate the performance of CNN based on increment of data samples, 3 axes torque values were considered during the training phase in order to make the prediction of forces more precise. The reference torque values were provided from an ATI F/T sensor whereas the 9 taxel solution of the sensor made the inclusion of torque values possible. An experimental validation of performance of sensors considering torque values were carried out for pushing and sliding actions of the thumb. Parameters and results obtained in this experimentation phase are given in Supplementary Table 5.

**FIGURE 11:**
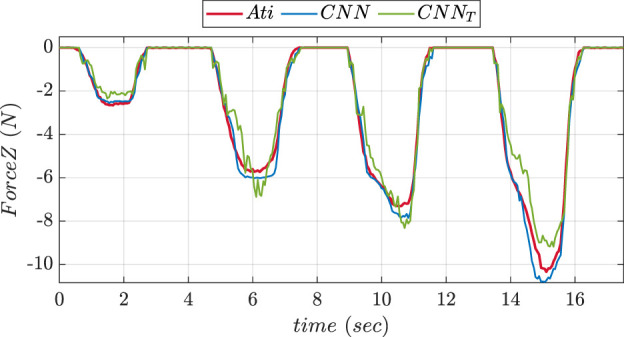
Comparative prediction graph of Z-axis force using CNN with torque (CNNt) and without torque (CNN) during push experiment.

**FIGURE 12:**
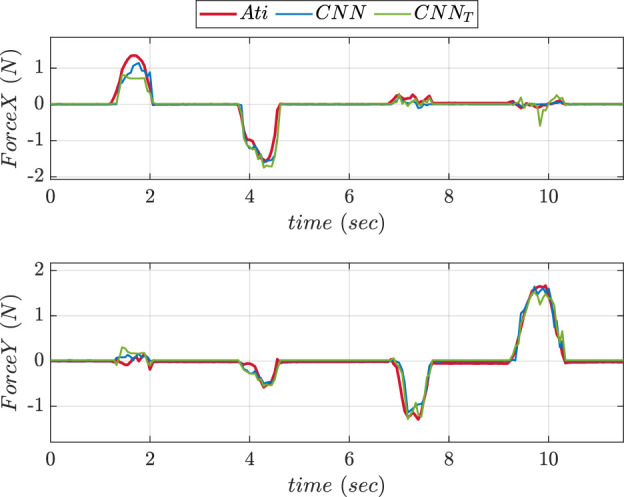
Comparative prediction graphs of X and Y-axes force using CNN with torque (CNNt) and without torque (CNN) during slide experiment.

The MSE values in X, Y, and Z directions during force training were found to be MSEx = 0.022, MSEy = 0.031 and MSEz = 0.400 respectively. The MSE values in X, Y, and Z directions during torque training were found to be MSEx = 9.932, MSEy = 8.841, and MSEz = 0.465 respectively. Figure 11 showcases a graph comparing the performance of prediction of *Z*-axis forces obtained using CNN with and without considering torque values during pushing and slidin experiments. Figure 12 shows graph comparing the performance of prediction of X and Y-axes forces using two CNN networks during sliding experiment.

The MSE values in Z-axis during pushing experiment were obtained as 0.11 for CNN without considering torque and 0.35 for CNN considering torque. The MSE values in X-axis during sliding experiment were found to be 0.013 for CNN without considering torque and 0.023 for CNN considering torque. The MSE values in Y-axis during sliding experiment were obtained as 0.01 for CNN without considering torque and 0.012 for CNN considering torque. MSE statistics showed that the accuracy of network reduced during force prediction when torque values were added to the CNN network.

#### 6.2.2 Inclusion of more layers in CNN training network

On the basis of the previous approach for the inclusion of torque during training, an increment of layers in the same model of CNN was made to improve the learning ability of the network. In this architecture, 5 convolutional layers were chosen with 128, 64, 32, 32, and 16 neurons. Furthermore, a dense layer of 16 neurons was added to obtain an output layer of 6 neurons including torque and force values. Supplementary Table 6 depicts the parameters of CNN and results obtained during this experimentation phase.

**FIGURE 13:**
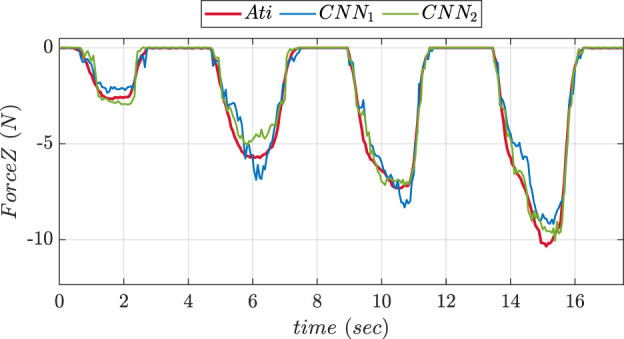
Comparative prediction graph of Z-axis force using CNN with less layers (CNN1) and more layers (CNN2) during push experiment.

**FIGURE 14:**
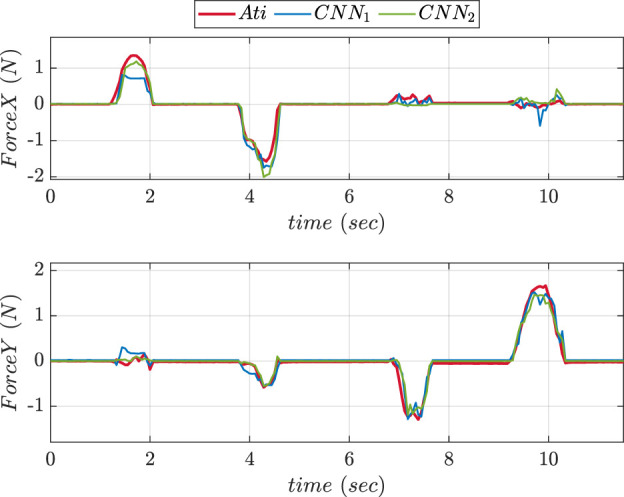
Comparative prediction graphs of X and Y-axes force using CNN with less layers (CNN1) and more layers (CNN2) during slide experiment.

**FIGURE 15:**
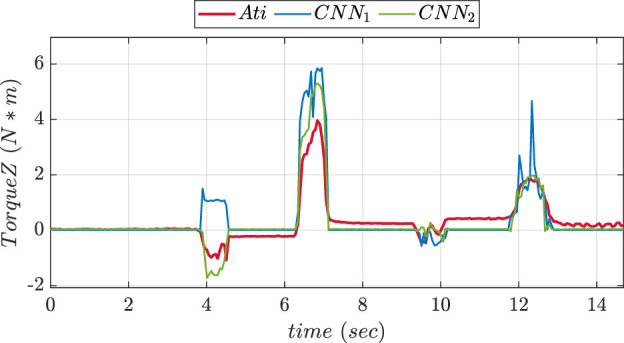
Experiment 2 using torque: Comparative prediction graph of Z-axis torque using CNN with less layers (CNN1) and more layers (CNN2).

**FIGURE 16:**
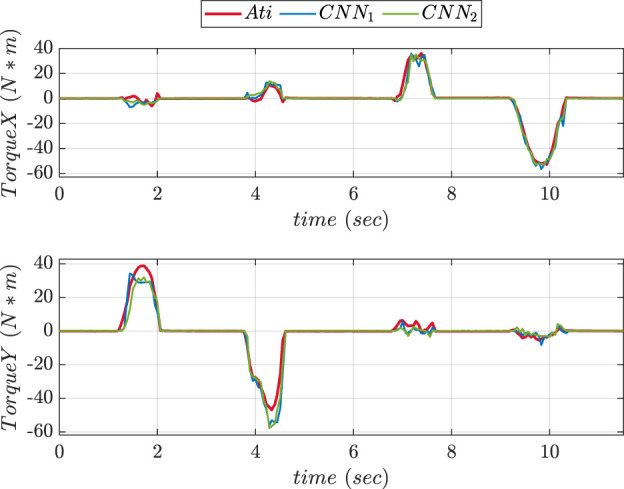
Experiment 1 using torque: Comparative prediction graphs of X and Y-axes torque using CNN with less layers (CNN1) and more layers (CNN2).

An experimental comparison for evaluating the precision of prediction for both CNN trained was done during pushing and sliding experiments. We consider CNN1 (older version) to be with fewer layers while CNN2 (updated version) to be with more layers. Figures 13, 14 show graphs depicting the performance of prediction of X, Y, and Z-axes forces during pushing and sliding experiments respectively. Moreover, experiments were also conducted in order to compare X, Y, and Z-axes torques using CNN with fewer layers (CNN1) and (CNN2). Graphs comparing the values of prediction are shown in Figures 15, 16. The MSE values for force training in X, Y, and Z directions were found to be MSEx = 0.022, MSEy = 0.043, and MSEz = 0.500 respectively. The MSE values for torque training in X, Y, and Z directions were obtained as MSEx = 11.302, MSEy = 9.221, and MSEz = 0.511 respectively.

The MSE values of prediction phase in Z-axis during push experiment were found to be 0.35 for CNN1 and 0.19 for CNN2. The MSE values of prediction phase in X-axis during slide experiment were obtained as 0.023 for CNN1 and 0.015 for CNN2. The MSE values of prediction phase in Y-axis during slide experiment were found to be 0.012 for CNN1 and 0.008 for CNN2. The MSE data showed that increasing the number of layers improved CNN performance in force prediction compared to fewer layers.

### 6.3 Experimental validations during in-hand manipulation tasks

**FIGURE 17:**
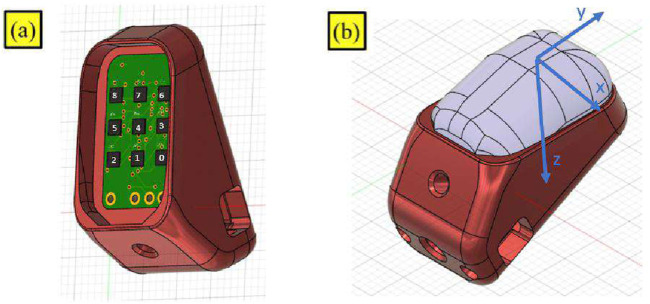
Sensor **(A)** Taxel numbering. **(B)** Sensor frame.

Experiments were conducted to determine the efficiency of the sensors to carry out in-hand manipulation tasks successfully. An object (a rubber ball) with unknown mass and geometry was chosen for performing the task. In the presented experiments, the manipulation approaches were based on the behavior of the taxels of the sensor placed on the thumb. Figure 17 displays the taxels equipped on the thumb sensors.

The condition for horizontal manipulation was based on the evaluation of taxels on the lateral side. The thumb moved horizontally in clockwise and anticlockwise directions at a constant speed of 20 rpm. In order to maintain a safe contact during the whole manipulation process, the lateral side taxels of the sensor were checked. In case of horizontal manipulation, initial motion of the thumb was carried out in clockwise direction until the lateral taxels (6–3–0) lost contact or it reached joint limits. After the initial phase of manipulation, the thumb was turned in an anticlockwise manner to return the object to its original position.

**FIGURE 18:**
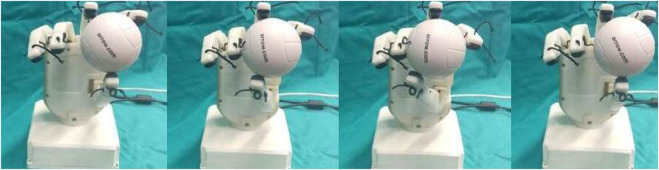
Frames during the horizontal manipulation phase.

**FIGURE 19:**
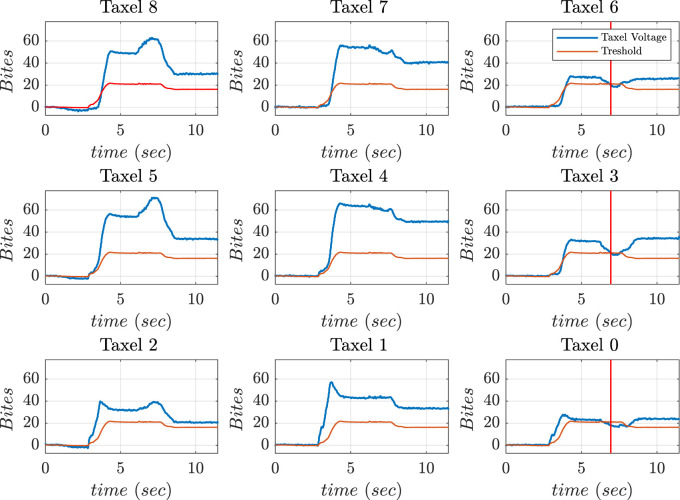
Voltage values of thumb sensor taxels along with threshold and instant of danger (red) indicated during horizontal manipulation phase.

Figure 18 shows the frames of horizontal manipulation using thumb. Figure 19 depicts the time instant of non-feasible contact on the graph when the left side taxel voltages fall under the threshold value (the reader should consider the mirror view of the taxel matrix in the figure). Figure 19 demonstrates the stop condition of clockwise manipulation at time instant 6.9 s when the voltage values of taxels (6–3–0) fall below the threshold. This signified that the right lateral side of the taxels is no longer in touch with the object. This circumstance increased the chance that the object was fall out of the hand hold.

The condition of vertical manipulation was based on behavior of the top and bottom taxels of the sensor presented on the thumb. Taking reference from Figure 17, the vertical manipulation comprised of 2 phases. The first phase of downward manipulation consisted of fingers closure and thumb extension in downward direction. Motions were carried out with a constant velocity of 10 rpm until the top taxels (6–7–8) lost contact or reached joint limits. Similarly, for upward manipulation, fingers opening and thumb flexion were carried out at a constant velocity of 10 rpm until the bottom taxels (0–1–2) break contact or the joint limits were reached.

**FIGURE 20:**
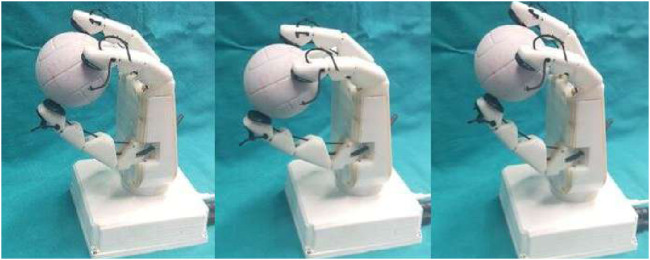
Frames during the vertical manipulation phase.

**FIGURE 21:**
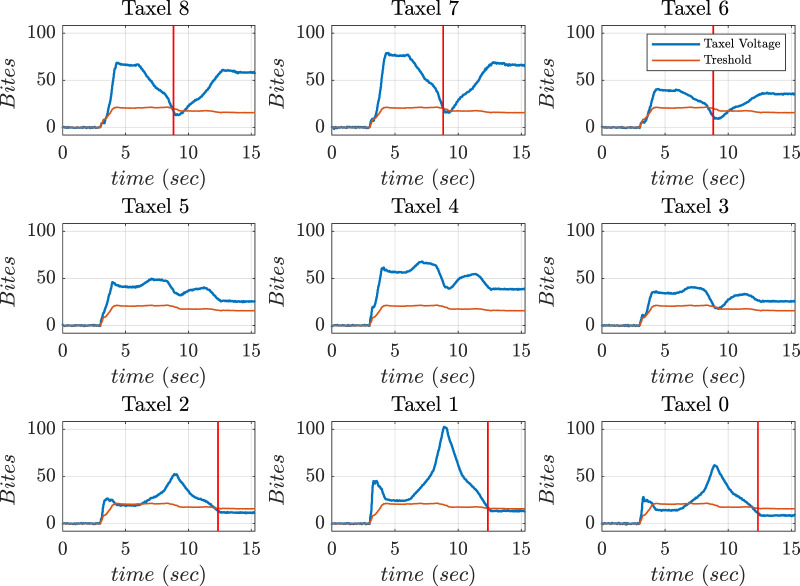
Voltage values of thumb sensor taxels along with threshold and instant of danger (red) indicated during vertical manipulation phase.

Figure 20 shows the screenshots of motions of fingers and thumb during vertical manipulation task. Figure 21 depicts graphs obtained during vertical manipulation tasks. The results showed that there were two stop time instants during the task. A stop time instant of downward manipulation at 8.8 s and stop time instant of upward manipulation at 12.3 s were detected from the results.

#### 6.3.1 Experimental analysis to check reaction to disturbance

A feasible manipulation of object was accomplished in the presence of external disturbances based on the voltage information. In this experiment, hand executed a pinching grasp and the controller determined a possibility of non-feasible grasps using instantaneous threshold value at each sampling time. The reaction to external disturbances during task execution was provided by taxels placed on the lateral side of the thumb. The thumb moved in the required direction (clockwise and anticlockwise) at a constant velocity of 20 rpm depending on which lateral side lost contact with the object. The rotation stopped when at least two taxels returned to threshold values. K was set to a value higher than 0.6 to increase the performance of the controller.

**FIGURE 22:**
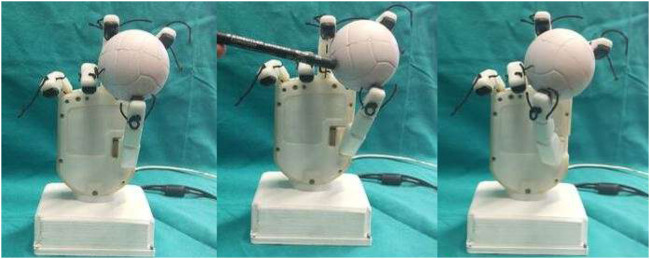
Frames during application of external disturbance.

**FIGURE 23:**
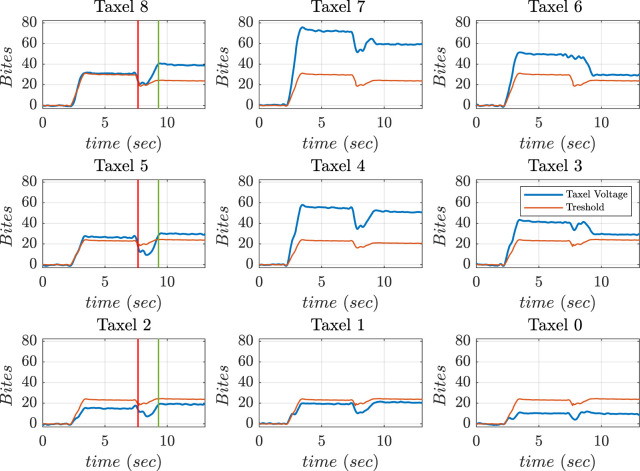
Voltage values of thumb sensor taxels, along with threshold (red), during external disturbance.

The hand grasped a foam ball and an external disturbance was applied to the ball using a pen as shown in Figure 22. The application of external disturbance caused a non-feasible left-side contact. Taxels such as 8, 5, and 2 fall below the threshold value. The motions were implemented to bring voltage outputs of taxels 8 and 5 inside threshold level. The disturbance caused on left-side was identified at time instant 7.9 (red) and a feasible contact was restored at time instant 9.2 (green) as shown in Figure 23.

### 6.4 Experiment to validate force control scheme while grasping

Force control enabled a variety of gripping techniques, including pinch, power, and lateral grasps, to be used securely and effectively to grasp objects with varying shapes and orientations. The Z-axis forces were considered during grasping tasks to determine threshold force. Similarly, forces in X and Y-axes were considered for computing threshold forces during slip conditions. The orientation of the axis of force on the fingertip pad are shown in Figure 17.

**FIGURE 24:**
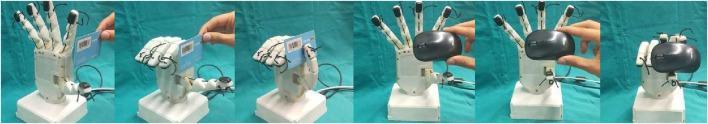
Frames of Lateral grasp and Pinch grasp.

**FIGURE 25:**
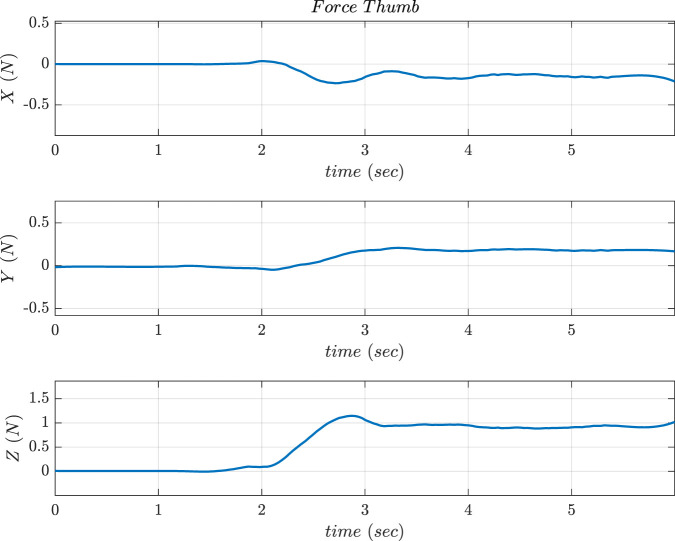
Force output obtained in 3 axes of Thumb sensor during Pinch Grasps (similar graphs are obtained for the lateral grasp).

Experiments for validating the force control scheme based on threshold values during grasping tasks were conducted. The motor moved at a speed of 60 rpm until a desired value of force was reached. The fingers and thumb rotations were set to a speed of 60 rpm. The stop criterion was ensured based on the value of force threshold in 2 phases for efficient grasping. The first phase was executed after the normal Z-axis force reached 0.8 N to stop the finger’s motion. Moreover, the second phase involved the stopping of the thumb flexion when the force reached a value of 1 N. An added advantage of this kind of approach was that the force limits can be modified easily to a higher or lower value depending on the requirement of grasping. Figure 24 shows the screenshots of motions of the hand during lateral and pinch grasps execution. Figure 25 shows the Z-axis force reaching a desired value of 1 N during pinch grasp (mouse). When the hand grasped an object laterally at different orientations, the motor that controlled the thumb and fingers moved them to predetermined places. In addition, the thumb moved at a speed of 60 rpm until the required force was applied.

### 6.5 Experimental validation during manipulation tasks combining raw tactile voltage and reconstructed force data

The predetermined force values were used for enhancing in-hand manipulation tasks. The performance of force controller described in Section 6.4 was improved using a predetermined force. The computed force values were used to exert desired grasping force to reduce the effects of external disturbance and restore a feasible grasp. The behavior of force in the *Z*-axis during the task is depicted in Supplementary Figure 1. The value of force reached around 1 N at time 4.2 s during in-hand manipulation of the object when an external disturbance force was applied. In order to maintain a stable grasp, 1 N force was applied to the hand in the opposite direction. The red line in Supplementary Figure 1 indicates the non feasible grasp. The restoration of the feasible grasp is indicated in yellow line. The restoration of required force (equal to 1 N) in *Z*-axis to obtain a feasible grasp is indicated by the green line. The values of raw tactile voltage values were used for carrying out the grasping task during the manipulation phase depicted between the red and the yellow lines. The in hand manipulation tasks were controlled using the combination of raw tactile voltage and reconstructed force values.

## 7 Conclusion and future works

This work presented a novel sensing strategy based on opto-electronic technology developed for a prosthetic hand. Grasping forces and torques were determined from voltages obtained from the novel sensor using ANN and CNN training methods. To summarize the performance of various trained NNs, the optimum approach for carrying out both pushing and slipping tasks was to use CNN compared to a ANN. The CNN2 was found to be the best model for determining force and torque values from ATI reference sensor measurements with better prediction accuracy, lower training and validation losses, better learning rate, and lower MSE values compared to CNN1. However, when torque values were introduced to the CNN network, the accuracy of the network decreased while predicting forces/torques.

Experimental evaluations on proposed novel sensor in this work demonstrated a force capacity upto 30 N in the Z-axis frame, which was sufficient for carrying out power, pinch, and lateral grasps. For force values less than 1.2 N, the precision of force prediction using NN differs by no more than 0.15 N from the actual force value. This disparity can be reduced by using an autonomous calibration procedure at the micro-force level to improve the sensor’s efficiency. The performance of the developed sensor was satisfactory when compared with the similar tactile sensors available in the market. The sensor could able to achieve a better sensing capability even though it is designed with dimensional constraints to place on the fingertips.

A method for controlling in-hand manipulation was developed in order to facilitate the grasping of objects with the hands in presence of external disturbances. Grasp forces were calculated by the control strategy using the reconstructed sensor data. The method employed taxel voltage data to regulate the object’s orientation. The adoption of control strategy to assist during grasping tasks enhanced the grasping of object without loosing contact even in the presence of external disturbances in vertical and horizontal directions. Experiments were conducted to validate the proposed strategies and analyze their effectiveness. The idea of in-hand manipulation control proposed in this work can be useful in the employment of the PRISMA Hand II in prosthetic as well as in the industrial domains. Future work will focus on employing the proposed force control strategy on all the fingers to improve efficiency of in-hand manipulation tasks.

## Data Availability

The original contributions presented in the study are included in the article/Supplementary Material, further inquiries can be directed to the corresponding author.
