# Supplementary material for: Validations of various in-hand object manipulation strategies employing a novel tactile sensor developed for an under-actuated robot hand
Source: Front Robot AI. 2024 Sep 26;11:1460589. doi: 10.3389/frobt.2024.1460589 (PMC11464259; doi:10.3389/frobt.2024.1460589)
Supplement: Supplementary file 1 [file DataSheet1.pdf]

Type of sensor	Robotic systems	Res./Rate
**Capacitive sensors**		
PPS RoboTouch [Cheng et al. (2016)]	Allegro robotic hand	25 $mm^2/30-100HZ$
PPS RoboTouch[Bogue (2019)]	Barrett hand	5 mm/30-100 HZ
**Optical tactile sensors**		
FBG sensor [Heo et al. (2008)]	Robotic finger	5mm/1.167 MHz
MBOF sensors[Heo et al. (2008)]	Robotic finger	5mm/20-25 KHZ
Soft viscoelastic skin[Yamada et al. (2005)]	Robotic finger	6mm/25 KHZ
Optoforce [Gregor et al. (2021)]	Barret hand	10 mm/20 HZ
**LED based tactile sensors**		
LED based sensor[Rossiter and Mukai (2005)]	Robotic finger	20 mm/ 1 KHZ
LED based sensor[Ohmura et al. (2006)]	Robotic finger	20 mm/ 20 MHZ
LED based sensor[Hoshino and Mori (2008)]	Robotic finger	0.1 mm/ 1 KHZ
**haptic sensors**		
LED based sensor[Sato et al. (2008)]	Robotic finger	5 mm/ 67 fps
**Piezoresistive tactile sensors**		
Tekscan[Liu et al. (2012b)]	Shadow hand	0.6mm/100 HZ
Rubber-based [Teshigawara et al. (2011)]	3-fingered hand	3 mm/10 KHZ
3D-shaped sensor [Koiva et al. (2013)]	Shadow hand	5.5 mm/1 KHZ
Rubber-based [Fukui et al. (2011)]	Universal robot hand	3.6 mm/50 HZ
Gifu hand sensor[Kawasaki et al. (2002)]	Gifu hand III	4 mm/10 HZ
Tekscan [Hollinger and Wanderley (2006)]	Shadow hand	4 mm/200 HZ
FSR [Kyberd and Chappell (1992)]	Southampton hand	6.5 mm/50 HZ
ATi Nano17 sensors [Wang et al. (2011)]	Shadow hand	5.5 mm/833 HZ
Weiss Robotics [Kappassov et al. (2015)]	Fluidic FRH-4 hand	3.5mm/230 fps
**Piezoelectric sensors**		
PRes. + PVDF [Goger et al. (2009)]	8 DoF fluid hand	3.5 mm/1 KHZ
PRes. + PVDF [Choi et al. (2006)]	SKKU hand I	0.5 mm/4 KHZ
Tactile skin [Strohmayr and Schneider (2013)]	DLR hand	1mm/20-50 HZ
**Barometric measurements based sensor**		
Takktile (silicon) [Ades et al. (2018)]	iHY robot hand	5 mm/50 HZ
BioTac (liquid) [Fishel et al. (2008)]	Shadow hand	5.5 mm/1 KHZ

Supplementary Table 1. Tactile sensors used in robotic hands

Type of sensor	Robotic systems	Learning method
Vision based sensor	Robotic finger	CNN[Kakani et al. (2021)]
Weiss Robotics + PVDF	Fluidic hand	kNN[Dang and Allen (2014)]
Weiss sensor	Schunk gripper and SDH hand	kNN [Drimus et al. (2014)]
PVDF + conductive foam	Fluid hand [70]	kNN [Goger et al. (2009)]
Ati Nano 17	Barret hand	kNN [Liu et al. (2012c)]
Digital accelerometer	STFT	kNN, SVM [Sinapov et al. (2011)]
Piezoresistive [58]	Schunk 3-finger hand [109]	SVM [Bekiroglu et al. (2011)]
BioTac sensor [84]	Shadow hand FFT,	SVM[Xu et al. (2013)]
Capacitive sensor arrays	Barret hand	SVM [Dang and Allen (2014)]
BioTac [84]	Shadow hand	ANN[Xu et al. (2013)]
PVDF based	Robotis motors fingers	decision trees[Jamali and Sammut (2011)]
Tekscan [57]	Shadow hand	Neural networks [Liu et al. (2012a)]
Tekscan TM(4256 E)	Barret hand PCA	Naive Bayes classifier [Liu et al. (2012d)]
BioTac sensor [84]	Robotic finger	Bayes[Fishel and Loeb (2012)]
Macroscale electronic skin	Schunk gripper	DNN [Sohn et al. (2017)]
GelSight Tactile Sensor	Robotic gripper	DNN[Yuan et al. (2017)]
Barometric sensor	Robotic two fingered gripper	Random forest[Spiers et al. (2016)]

Supplementary Table 2. Learning methods employed in robotic hands to reconstruct data from tactile sensors

Parameters	Simulation study	Experimentation	
		Pushing	sliding
Batch size	64	64	64
Epochs	300	300	300
Gradient	0.027	0.129	0.195
momentum	0.99	0.99	0.99
Weight decay	0.034	0.021	0.12
Mean square error in X direction	0.053	-	0.016
Mean square error in Y direction	0.049	-	0.024
Mean square error in Z direction	0.319	0.2164	-
Regression	0.89	0.87	0.85
Learning rate	0.001	0.001	0.001
Accuracy	0.72	0.70	0.67
Training loss	0.21	0.38	0.42
Validation loss	0.17	0.35	0.40

Supplementary Table 3. ANN based training parameters and results

Parameters/results	Simulation study	Experimentation	
		Pushing	sliding
Neurons-conv 1	256	256	256
Neurons-conv 2	64	64	64
Neurons-Dense layer	16	16	16
Kernel size- conv layers	3 x 3	3 x 3	3 x 3
Kernel size-pooling	2 x 2	2 x 2	2 x 2
Padding	0.319	0.053	0.053
Stride-conv layers	1	1	1
Stride-pooling	2	2	2
Mean square error in X direction	0.022	-	0.013
Mean square error in Y direction	0.022	-	0.010
Mean square error in Z direction	0.148	0.101	-
Learning rate	0.0001	0.0001	0.0001
Accuracy	0.98	0.93	0.91
Training loss	0.15	0.21	0.27
Validation loss	0.13	0.15	0.19

Supplementary Table 4. CNN based training parameters and results

Parameters	Force training	Including torque
Mean square error in X direction	0.022	9.932
Mean square error in Y direction	0.031	8.841
Mean square error in Z direction	0.400	0.465
Learning rate	0.0001	0.01
Accuracy	0.91	0.77
Training loss	0.24	0.35
Validation loss	0.11	0.253
MSE during sliding -X axis	0.013	0.023
MSE during pushing -Y axis	0.01	0.012
MSE during pushing -Z axis	0.11	0.35

Supplementary Table 5. CNN based training parameters including torque data

Parameters	CNN1	CNN2
Mean square error in X direction	0.023	0.015
Mean square error in Y direction	0.012	0.008
Mean square error in Z direction	0.35	0.19
Learning rate	0.0001	0.00001
Accuracy	0.93	0.98
Training loss	0.22	0.18
Validation loss	0.19	0.13

Supplementary Table 6. CNN1 and CNN2 based training parameters and results

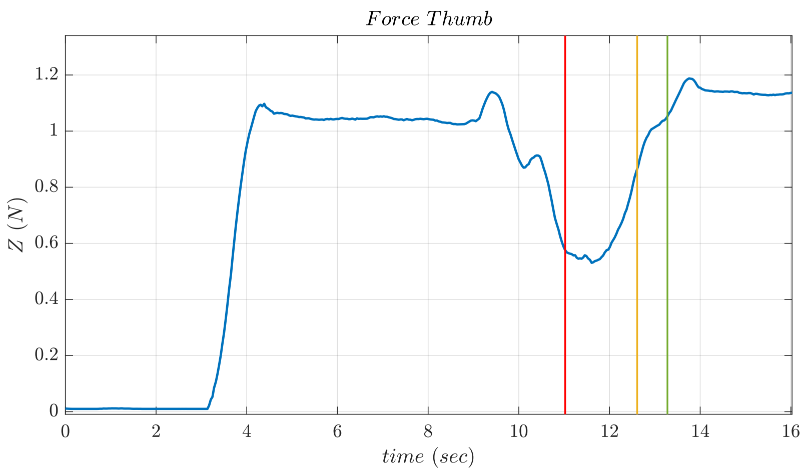

Supplementary Figure 1. Force output on Z axis during the whole process of grasping followed by in-hand manipulation and final force control.

REFERENCES

Ades, C., Gonzalez, I., AlSaidi, M., Nojoumian, M., Bai, O., Aravelli, A., et al. (2018). Robotic finger force sensor fabrication and evaluation through a glove. In *Proceedings. Florida Conference on Recent Advances in Robotics* (NIH Public Access), vol. 2018, 60

Bekiroglu, Y., Laaksonen, J., Jorgensen, J. A., Kyrki, V., and Kragic, D. (2011). Assessing grasp stability based on learning and haptic data. *IEEE Transactions on Robotics* 27, 616–629

Bogue, R. (2019). Tactile sensing for surgical and collaborative robots and robotic grippers. *Industrial Robot: the international journal of robotics research and application* 46, 1–6

Cheng, D., Dong, S., Wang, X., and Jin, H. (2016). Stretchable tiny stress tactile sensor based on capacitor array. In *2016 IEEE International Nanoelectronics Conference (INEC)* (IEEE), 1–2

Choi, B., Lee, S., Choi, H. R., and Kang, S. (2006). Development of anthropomorphic robot hand with tactile sensor: Skku hand ii. In *2006 IEEE/RSJ International Conference on Intelligent Robots and Systems* (IEEE), 3779–3784

Dang, H. and Allen, P. K. (2014). Stable grasping under pose uncertainty using tactile feedback. *Autonomous Robots* 36, 309–330

Drimus, A., Kootstra, G., Bilberg, A., and Kragic, D. (2014). Design of a flexible tactile sensor for classification of rigid and deformable objects. *Robotics and Autonomous Systems* 62, 3–15

Fishel, J. A. and Loeb, G. E. (2012). Bayesian exploration for intelligent identification of textures. *Frontiers in neurorobotics* 6, 4

Fishel, J. A., Santos, V. J., and Loeb, G. E. (2008). A robust micro-vibration sensor for biomimetic fingertips. In *2008 2nd IEEE RAS & EMBS International Conference on Biomedical Robotics and Biomechatronics* (IEEE), 659–663

Fukui, W., Kobayashi, F., Kojima, F., Nakamoto, H., Imamura, N., Maeda, T., et al. (2011). High-speed tactile sensing for array-type tactile sensor and object manipulation based on tactile information. *Journal of Robotics* 2011, 691769

Goger, D., Gorges, N., and Worn, H. (2009). Tactile sensing for an anthropomorphic robotic hand: Hardware and signal processing. In *2009 IEEE International Conference on Robotics and Automation* (IEEE), 895–901

Gregor, R., Babinec, A., Duchoň, F., and Dobiš, M. (2021). Hand guiding a virtual robot using a force sensor. *acta mechanica et automatica* 15, 177–186

Heo, J.-S., Kim, J.-Y., and Lee, J.-J. (2008). Tactile sensors using the distributed optical fiber sensors. In *2008 3rd International Conference on Sensing Technology* (IEEE), 486–490

Hollinger, A. and Wanderley, M. M. (2006). Evaluation of commercial force-sensing resistors. In *Proceedings of the International Conference on New Interfaces for Musical Expression, Paris, France* (Citeseer), 4–8

Hoshino, K. and Mori, D. (2008). Three-dimensional tactile sensor with thin and soft elastic body. In *2008 IEEE Workshop on Advanced robotics and Its Social Impacts* (IEEE), 1–6

Jamali, N. and Sammut, C. (2011). Majority voting: Material classification by tactile sensing using surface texture. *IEEE Transactions on Robotics* 27, 508–521

Kakani, V., Cui, X., Ma, M., and Kim, H. (2021). Vision-based tactile sensor mechanism for the estimation of contact position and force distribution using deep learning. *Sensors* 21, 1920

Kappassov, Z., Corrales, J.-A., and Perdereau, V. (2015). Tactile sensing in dexterous robot hands. *Robotics and Autonomous Systems* 74, 195–220

Kawasaki, H., Komatsu, T., and Uchiyama, K. (2002). Dexterous anthropomorphic robot hand with distributed tactile sensor: Gifu hand ii. *IEEE/ASME transactions on mechatronics* 7, 296–303

Koiva, R., Zenker, M., Schürmann, C., Haschke, R., and Ritter, H. J. (2013). A highly sensitive 3d-shaped tactile sensor. In *2013 IEEE/ASME International Conference on Advanced Intelligent Mechatronics* (IEEE), 1084–1089

Kyberd, P. J. and Chappell, P. H. (1992). Object-slip detection during manipulation using a derived force vector. *Mechatronics* 2, 1–13

Liu, H., Greco, J., Song, X., Bimbo, J., Seneviratne, L., and Althoefer, K. (2012a). Tactile image based contact shape recognition using neural network. In *2012 IEEE International Conference on Multisensor Fusion and Integration for Intelligent Systems (MFI)* (IEEE), 138–143

Liu, H., Song, X., Bimbo, J., Althoefer, K., and Senerivatne, L. (2012b). Intelligent fingertip sensing for contact information identification. In *Advances in Reconfigurable Mechanisms and Robots I* (Springer), 599–608

Liu, H., Song, X., Bimbo, J., Seneviratne, L., and Althoefer, K. (2012c). Surface material recognition through haptic exploration using an intelligent contact sensing finger. In *2012 IEEE/RSJ international conference on intelligent robots and systems* (IEEE), 52–57

Liu, H., Song, X., Nanayakkara, T., Seneviratne, L. D., and Althoefer, K. (2012d). A computationally fast algorithm for local contact shape and pose classification using a tactile array sensor. In *2012 IEEE International Conference on Robotics and Automation* (IEEE), 1410–1415

Ohmura, Y., Kuniyoshi, Y., and Nagakubo, A. (2006). Conformable and scalable tactile sensor skin for curved surfaces. In *Proceedings 2006 IEEE International Conference on Robotics and Automation, 2006. ICRA 2006.* (IEEE), 1348–1353

Rossiter, J. and Mukai, T. (2005). A novel tactile sensor using a matrix of leds operating in both photoemitter and photodetector modes. In *SENSORS, 2005 IEEE* (IEEE), 4–pp

Sato, K., Kamiyama, K., Nii, H., Kawakami, N., and Tachi, S. (2008). Measurement of force vector field of robotic finger using vision-based haptic sensor. In *2008 IEEE/RSJ International Conference on Intelligent Robots and Systems* (IEEE), 488–493

Sinapov, J., Sukhoy, V., Sahai, R., and Stoytchev, A. (2011). Vibrotactile recognition and categorization of surfaces by a humanoid robot. *IEEE Transactions on Robotics* 27, 488–497

Sohn, K.-S., Chung, J., Cho, M.-Y., Timilsina, S., Park, W. B., Pyo, M., et al. (2017). An extremely simple macroscale electronic skin realized by deep machine learning. *Scientific reports* 7, 11061

Spiers, A. J., Liarokapis, M. V., Calli, B., and Dollar, A. M. (2016). Single-grasp object classification and feature extraction with simple robot hands and tactile sensors. *IEEE transactions on haptics* 9, 207–220

Strohmayr, M. and Schneider, D. (2013). The dlr artificial skin step ii: Scalability as a prerequisite for whole-body covers. In *2013 IEEE/RSJ International Conference on Intelligent Robots and Systems* (IEEE), 4721–4728

Teshigawara, S., Tsutsumi, T., Shimizu, S., Suzuki, Y., Ming, A., Ishikawa, M., et al. (2011). Highly sensitive sensor for detection of initial slip and its application in a multi-fingered robot hand. In *2011 IEEE International Conference on Robotics and Automation* (IEEE), 1097–1102

Wang, D., Fang, L., Xu, M., and Yu, J. (2011). Flexible building blocks: Modularized design concept of a six-axis force/torque sensor. In *2011 IEEE International Conference on Mechatronics and Automation* (IEEE), 1109–1114

Xu, D., Loeb, G. E., and Fishel, J. A. (2013). Tactile identification of objects using bayesian exploration. In *2013 IEEE international conference on robotics and automation* (IEEE), 3056–3061

Yamada, Y., Morizono, T., Umetani, Y., and Takahashi, H. (2005). Highly soft viscoelastic robot skin with a contact object-location-sensing capability. *IEEE Transactions on Industrial electronics* 52, 960–968

Yuan, W., Zhu, C., Owens, A., Srinivasan, M. A., and Adelson, E. H. (2017). Shape-independent hardness estimation using deep learning and a gelsight tactile sensor. In *2017 IEEE International Conference on Robotics and Automation (ICRA)* (IEEE), 951–958
